# UNDERSTANDING STROKE SURVIVOR–CARER DYNAMICS USING THE ACTOR-PARTNER INTERDEPENDENCE MODEL: A SYSTEMATIC REVIEW

**DOI:** 10.1093/geroni/igac059.2351

**Published:** 2022-12-20

**Authors:** Esther Chow, Sai-fu Fung

**Affiliations:** City University of Hong Kong, Kowloon, Hong Kong; City University of Hong Kong, Kowloon, Hong Kong

## Abstract

**Background:**

Family members often take on the caregiver roles to stroke survivors due to kinships and cultural responsibilities. Yet, little is known about the the impact of survivor-caregiver dynamics on stroke rehabilitation. To understand the dyadic relationships between the dyads, a systematic review was conducted to examine studies which have adopted Actor–Partner Interdependence Model (APIM) analysis.

**Methods:**

A systematic review and meta-analysis were conducted using the following electronic databases: PubMed, SAGE Journals, MEDLINE, PsycINFO and Cochrane Review to identify eligible studies published from their inception to December 2021, following the PRISMA guidelines.

**Results:**

Nine studies involving 1183 stroke survivors (male = 57%) and 1181 caregivers (male = 37%) had met the inclusion criteria and were identified in this review. The interaction among the following outcomes were self-esteem, optimism, stress, depression, emotional distress, quality of life, and life satisfaction. The review provided evidence that (1) APIM is valuable tool in analyzing dyadic interactions; (2) support from caregivers has a significant impact on the stroke survivor’s recovery; (3) the mental health status of caregivers can influence that of stroke survivors and vice versa; (4) Due to the interconnected nature of dyadic interaction, providing dyadic intervention have positive impact on dyads.

**Conclusion:**

These findings highlight the interdependence nature between survivor-caregiver dyads in the context of stroke rehabilitation. The APIM provides conclusive evidence on the effectiveness of survivor-caregiver dyadic interactions, with significant theoretical and practice implications for both health and social care professions. More research is needed to support dyadic strategies for stroke survivors.

